# Cell Adhesion Molecules Involved in Neurodevelopmental Pathways Implicated in 3p-Deletion Syndrome and Autism Spectrum Disorder

**DOI:** 10.3389/fncel.2020.611379

**Published:** 2021-01-13

**Authors:** Josan Gandawijaya, Rosemary A. Bamford, J. Peter H. Burbach, Asami Oguro-Ando

**Affiliations:** ^1^University of Exeter Medical School, University of Exeter, Exeter, United Kingdom; ^2^Department of Translational Neuroscience, Brain Center Rudolf Magnus, University Medical Center Utrecht and Utrecht University, Utrecht, Netherlands

**Keywords:** 3p-deletion syndrome, autism spectrum disorder, copy number variation, IgCAM, neurogenesis, axon guidance, synaptic plasticity

## Abstract

Autism spectrum disorder (ASD) is characterized by impaired social interaction, language delay and repetitive or restrictive behaviors. With increasing prevalence, ASD is currently estimated to affect 0.5–2.0% of the global population. However, its etiology remains unclear due to high genetic and phenotypic heterogeneity. Copy number variations (CNVs) are implicated in several forms of syndromic ASD and have been demonstrated to contribute toward ASD development by altering gene dosage and expression. Increasing evidence points toward the p-arm of chromosome 3 (chromosome 3p) as an ASD risk locus. Deletions occurring at chromosome 3p result in 3p-deletion syndrome (Del3p), a rare genetic disorder characterized by developmental delay, intellectual disability, facial dysmorphisms and often, ASD or ASD-associated behaviors. Therefore, we hypothesize that overlapping molecular mechanisms underlie the pathogenesis of Del3p and ASD. To investigate which genes encoded in chromosome 3p could contribute toward Del3p and ASD, we performed a comprehensive literature review and collated reports investigating the phenotypes of individuals with chromosome 3p CNVs. We observe that high frequencies of CNVs occur in the 3p26.3 region, the terminal cytoband of chromosome 3p. This suggests that CNVs disrupting genes encoded within the 3p26.3 region are likely to contribute toward the neurodevelopmental phenotypes observed in individuals affected by Del3p. The 3p26.3 region contains three consecutive genes encoding closely related neuronal immunoglobulin cell adhesion molecules (IgCAMs): *Close Homolog of L1* (*CHL1*), *Contactin-6* (*CNTN6*), and *Contactin-4* (*CNTN4*). CNVs disrupting these neuronal IgCAMs may contribute toward ASD phenotypes as they have been associated with key roles in neurodevelopment. CHL1, CNTN6, and CNTN4 have been observed to promote neurogenesis and neuronal survival, and regulate neuritogenesis and synaptic function. Furthermore, there is evidence that these neuronal IgCAMs possess overlapping interactomes and participate in common signaling pathways regulating axon guidance. Notably, mouse models deficient for these neuronal IgCAMs do not display strong deficits in axonal migration or behavioral phenotypes, which is in contrast to the pronounced defects in neuritogenesis and axon guidance observed *in vitro*. This suggests that when CHL1, CNTN6, or CNTN4 function is disrupted by CNVs, other neuronal IgCAMs may suppress behavioral phenotypes by compensating for the loss of function.

## Introduction

### Autism Spectrum Disorder

Autism spectrum disorder (ASD) describes a group of neurodevelopmental disorders characterized by three core behavior domains: deficits in social interaction, delayed language development and repetitive or restrictive behaviors (American Psychiatric Association, 2013). ASD is currently estimated to affect 0.5–2.0% of the global population, with ever-increasing prevalence (Zablotsky et al., 2015; World Health Organization, 2019; Centers for Disease Control and Prevention, 2020; Maenner et al., 2020; May et al., 2020; Shaw et al., 2020). In addition to its phenotypic heterogeneity, several studies have reported associations between ASD and other neurodevelopmental and neuropsychiatric disorders, including but not limited to schizophrenia (Zheng et al., 2018; De Crescenzo et al., 2019), epilepsy (Besag, 2018), and major depressive disorder (Magnuson and Constantino, 2011). Individuals with ASD frequently present with intellectual and learning disabilities, demonstrating impaired cognitive function (Srivastava and Schwartz, 2014). Although ASD etiology remains unclear, several studies have demonstrated that ASD possesses a strong genetic component, with concordance rates in monozygotic twins as high as 95% (Abrahams and Geschwind, 2008; Sandin et al., 2014; Colvert et al., 2015; De La Torre-Ubieta et al., 2016; Ramaswami and Geschwind, 2018; Rylaarsdam and Guemez-Gamboa, 2019). The increased power of modern DNA sequencing technologies and number of ASD case-parent trio studies has allowed researchers to identify common inherited and rare *de novo* mutations that confer risk toward ASD development, and to date hundreds of ASD candidate genes have been reported (Rylaarsdam and Guemez-Gamboa, 2019).

### Copy Number Variations and Their Contribution to Autism Spectrum Disorder Genetics

**FIGURE 1:**
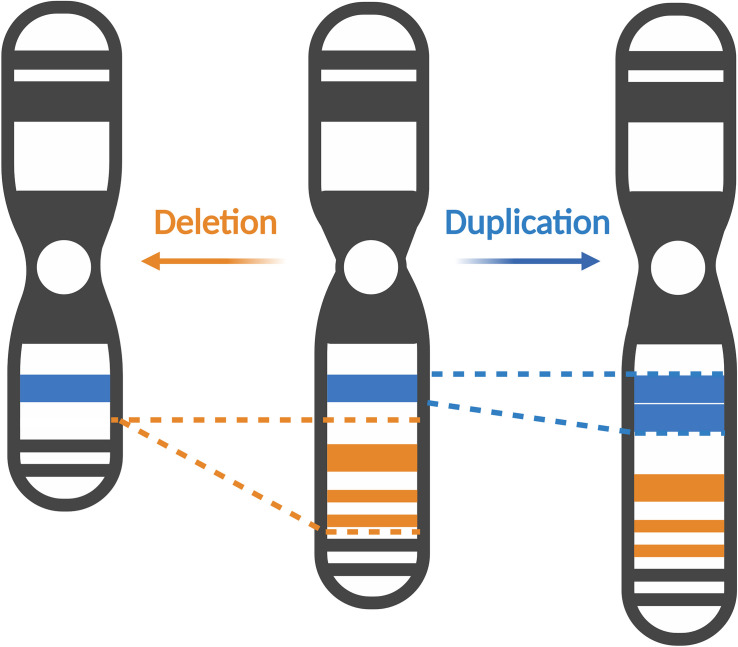
Copy number variations alter gene dosage and expression. Copy number variations (CNVs) can manifest as deletions, where segments of DNA are removed (represented in orange), or duplications, where extra copies of a DNA segment are produced (represented in blue). CNVs can vary in size, affecting several exons in a gene, to multiple genes or even cytobands of a chromosome. Created with BioRender.com.

Copy number variations (CNVs) are structural genetic changes whereby segments of DNA on a chromosome – usually defined as greater than 1 kb in size – become deleted or duplicated through erroneous DNA replication (Figure 1). CNVs can be inherited or arise *de novo* and alter gene dosage (i.e., the number of copies of alleles of a given gene) and expression (i.e., the amount of a gene transcribed as RNA or translated into protein) (Gamazon and Stranger, 2015). Large CNVs (greater than 1 Mb) were among the first types of *de novo* genetic variation discovered in ASD and more recently, smaller CNVs have been associated with ASD as well (Ramaswami and Geschwind, 2018). Notably, individuals with ASD are observed to carry significantly more CNVs than their unaffected siblings or control subjects (Sebat et al., 2007; Pinto et al., 2010; Levy et al., 2011; Leppa et al., 2016). Multiple CNVs occurring at several loci including chromosomes 7q, 15q, 16p, 22q, and others have been implicated in ASD (Jacquemont et al., 2006; Marshall et al., 2008; Bucan et al., 2009; Glessner et al., 2009; Ramaswami and Geschwind, 2018). These CNVs cause clinically defined syndromes that are often comorbid with ASD, so called “syndromic” forms of ASD. Studying these rare genetic syndromes and how they overlap with ASD phenotypes will reveal candidate genes and convergent molecular pathways linking various forms of ASD. In this review, we will focus on CNVs occurring in the p-arm of chromosome 3 (henceforth referred to as chromosome 3p) and how they contribute toward ASD risk.

### Copy Number Variations in Chromosome 3p26.3 as Risk Factors for ASD

**FIGURE 2:**
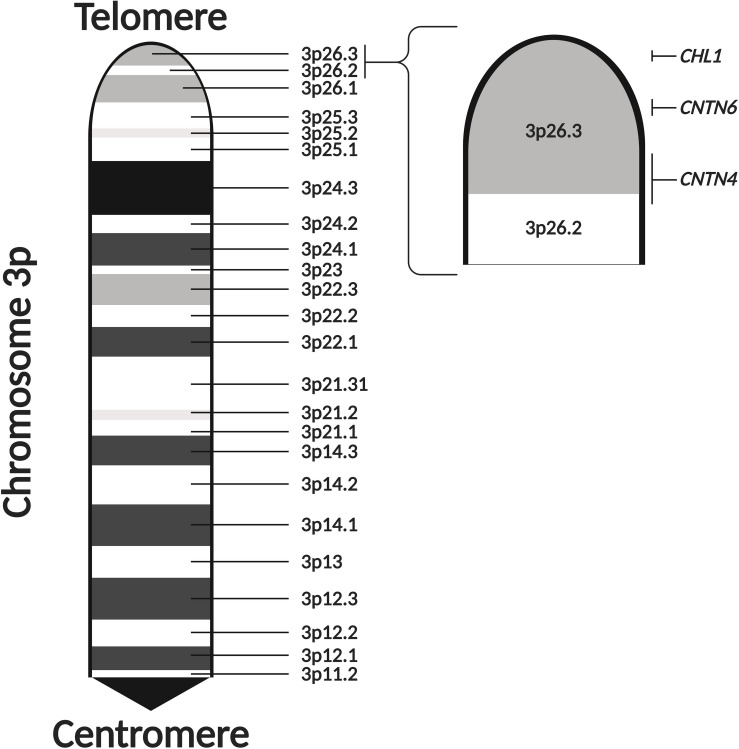
The 3p26.3 region encodes three neuronal cell adhesion molecules of the immunoglobulin superfamily. The 3p26.3 region is located at the distal end of the p-arm of chromosome 3. It is the terminal cytoband, lying just before the telomeres and containing three genes (in order from the telomere): *Close Homolog of L1* (*CHL1*), *Contactin-6* (*CNTN6*), and *Contactin-4* (*CNTN4*). Figure adapted from the *Ensembl Genome Browser* (Yates et al., 2020). Created with BioRender.com.

There is increasing evidence that CNVs occurring at chromosome 3p contribute toward ASD risk. Deletions at chromosome 3p result in 3p-deletion syndrome (Del3p), a rare genetic disorder characterized by developmental delay, intellectual disability, microcephaly, and various facial dysmorphisms and limb abnormalities (Verjaal and De Nef, 1978; Shuib et al., 2009; Peltekova et al., 2012; Kellogg et al., 2013). Cases of Del3p often present with neurodevelopmental and neuropsychiatric phenotypes, such as ASD, schizophrenia, and epilepsy (Schinzel, 1981; Drumheller et al., 1996; Fernandez et al., 2004; Bittel et al., 2006; Dijkhuizen et al., 2006; Malmgren et al., 2007; Roohi et al., 2009; Pohjola et al., 2010; Cottrell et al., 2011; Palumbo et al., 2013; Moghadasi et al., 2014; de la Hoz et al., 2015; Hu et al., 2015; Guo et al., 2017; Juan-Perez et al., 2018; Parmeggiani et al., 2018). Individuals affected by Del3p also tend to display neuroanatomical abnormalities as well (Malmgren et al., 2007; Pariani et al., 2009; Carr et al., 2010; Kellogg et al., 2013; Kuechler et al., 2015; Hajek et al., 2018; Parmeggiani et al., 2018). Although there are limited reports, duplications at chromosome 3p have been shown to present with ASD-associated phenotypes (Kashevarova et al., 2014; Li et al., 2016). Therefore, studying CNVs occurring in chromosome 3p could lead to the discovery of dosage-sensitive genes which are involved in molecular mechanisms that regulate neurodevelopmental processes. Genome-wide CNV studies in ASD cohorts have also detected ASD-specific and recurrent CNVs in the 3p26.3 cytoband, a region located at the distal end of the p-arm (Figure 2; Glessner et al., 2009; Levy et al., 2011; Guo et al., 2017). In a previous review of Del3p cases, Peltekova et al. (2012) noted that many of the cases of chromosome 3p CNVs that are comorbid with ASD occur at more distal cytobands of the p-arm. Furthermore, it is rare for individuals with a 3p26 deletion to display normal intelligence (Shuib et al., 2009). Therefore, it is likely that disruption of genes encoded in the 3p26.3 region play a role in the development of neurodevelopmental phenotypes in individuals with Del3p.

The 3p26.3 region contains three consecutive genes encoding closely related neuronal cell adhesion molecules of the immunoglobulin superfamily (IgCAMs), in order from the telomere: *Close Homolog of L1* (*CHL1*), *Contactin-6* (*CNTN6*), and *Contactin-4* (*CNTN4*) (Figure 2). Interestingly, the SFARI Gene database scores *CNTN6* and *CNTN4* as strong ASD candidate genes (Abrahams et al., 2013). These neuronal IgCAMs have been observed to play key roles in neurodevelopment, regulating axon guidance, neuronal migration and synaptic plasticity (Betancur et al., 2009; Sytnyk et al., 2017). Importantly, CNVs and other mutations affecting these neuronal IgCAMs have been reported in individuals with neurodevelopmental and neuropsychiatric phenotypes (Table 1). For example, CNVs in *CHL1* have been observed in individuals with ASD, intellectual disability and epilepsy, and are associated with speech delay, difficulties with expressive language, learning disabilities and cognitive impairment (Angeloni et al., 1999; Pohjola et al., 2010; Cuoco et al., 2011; Shoukier et al., 2013; Tassano et al., 2014; Li et al., 2016; Bitar et al., 2019; Table 1). CNVs in *CNTN6* have similarly been reported in individuals with ASD, schizophrenia, intellectual disability, bipolar disorder, and epilepsy (Kashevarova et al., 2014; Noor et al., 2014; Hu et al., 2015; Li et al., 2016; Mercati et al., 2017; Juan-Perez et al., 2018; Tassano et al., 2018; Repnikova et al., 2020; Table 1). Finally, CNVs involving *CNTN4* have been observed in individuals with ASD, language delay, and seizures as well (Fernandez et al., 2004; Fernandez T. et al., 2008; Fernandez T. V. et al., 2008; Dijkhuizen et al., 2006; Glessner et al., 2009; Roohi et al., 2009; Pinto et al., 2010; Cottrell et al., 2011; Poultney et al., 2013; Zhang et al., 2020; Table 1). These observations suggest that the cellular pathways regulated by these neuronal IgCAMs are important contributors to the behavioral phenotypes of neurodevelopmental disorders such as ASD. Therefore, it is imperative to obtain a greater understanding of the functions of these neuronal IgCAMs, their interactors and how they modulate the cellular pathways underlying ASD etiology.

**TABLE 1:** Overview of phenotypes observed in individuals with CNVs in *CHL1*, *CNTN6*, and *CNTN4*.

CNV in Gene	Phenotypes Observed	References
*CHL1*	Autism spectrum disorder	Angeloni et al., 1999; Pohjola et al., 2010; Cuoco et al., 2011; Shoukier et al., 2013; Tassano et al., 2014; Li et al., 2016; Bitar et al., 2019
	Epilepsy	
	Intellectual disability	
	Cognitive impairments and learning disabilities	
	Speech delay and language difficulties	

*CNTN6*	Autism spectrum disorder	Kashevarova et al., 2014; Noor et al., 2014; Hu et al., 2015; Li et al., 2016; Mercati et al., 2017; Juan-Perez et al., 2018; Tassano et al., 2018; Repnikova et al., 2020
	Epilepsy	
	Schizophrenia	
	Bipolar disorder	
	Intellectual disability	

*CNTN4*	Autism spectrum disorder Speech delay and language difficulties Seizures	Fernandez et al., 2004; Fernandez T. et al., 2008; Fernandez T. V. et al., 2008; Dijkhuizen et al., 2006; Glessner et al., 2009; Roohi et al., 2009; Pinto et al., 2010; Cottrell et al., 2011; Poultney et al., 2013; Zhang et al., 2020

*Further details on the coordinates and size of these CNVs, their *de novo* or inherited status and a more specific breakdown of behavior and developmental phenotypes can be found in Supplementary Table 1.*

Notably, CNVs in the 3p26.3 region show a spectrum of phenotypic severity, and demonstrate incomplete penetrance for ASD and other neurodevelopmental phenotypes (elaborated upon in section “CNVs in the 3p26.3 Region Are Associated With Neurodevelopmental Behavior Phenotypes” of this review). However, larger CNVs affecting two or all three neuronal IgCAMs encoded in the 3p26.3 region are observed to produce more severe phenotypes, suggesting that disruptions to multiple members of these neuronal IgCAMs exert additive effects that contribute to phenotype severity. This would also explain the incomplete penetrance commonly observed with CNVs that only affect one of these genes, and why severe behavior phenotypes have not been observed in animal knockout models for *Chl1* or *Cntn4* (discussed later in section “Behavior Phenotypes of Animal Models” of this review). As such, this review aims to discuss our current understanding of the functions of CHL1, CNTN6, and CNTN4, examining their overlapping expression patterns and interactomes during neurodevelopment. We will discuss how these genes regulate neuronal migration and neurite outgrowth, how this alters synaptogenesis and synaptic plasticity and ultimately, behavior phenotypes in animal models.

## Copy Number Variations in Chromosome 3p26.3

### CNVs in the 3p26.3 Region Are Associated With Neurodevelopmental Behavior Phenotypes

Using the online *PubMed* database, 195 studies investigating individuals with CNVs or chromosomal rearrangements in chromosome 3p were collected (Supplementary Table 1). In total, 894 individuals with a total of 907 CNVs were collated. Where available, CNV coordinates, properties (deletion or duplication), inheritance status, and ASD diagnoses were recorded (Supplementary Table 1). Additionally, any neurodevelopmental or neuropsychiatric phenotypes were also recorded, which included: the presence of autistic features (i.e., individuals displaying any or all of the core behavior domains of ASD but without an ASD diagnosis), Rett syndrome, Asperger syndrome, pervasive developmental disorder not otherwise specified (PDD-NOS), schizophrenia, Prader-Willi syndrome, attention deficit disorder or hyperactivity, bipolar disorder, intellectual disability, developmental delay, seizures or abnormal electroencephalogram, and any neuroanatomical abnormalities (as identified by magnetic resonance imaging) (Supplementary Table 1). Studies investigating chromosome 3p CNVs in cancerous tumors were excluded.

**FIGURE 3:**
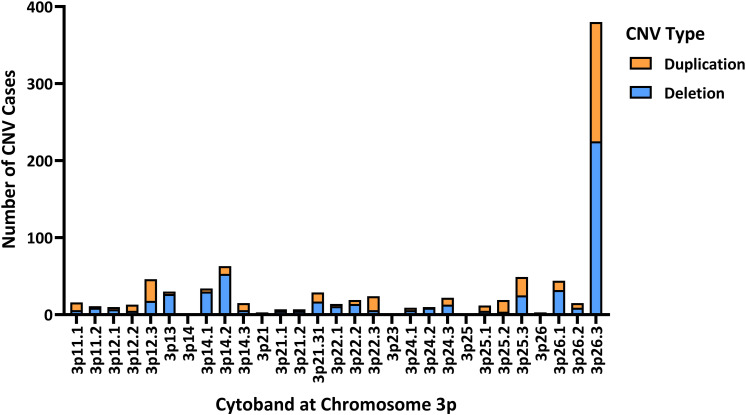
The distribution of CNVs at chromosome 3p mapped by their distal breakpoint. Most deletions and duplications reported at chromosome 3p occur within the 3p26.3 region (380 of 907 CNVs; consisting of 225 deletions and 155 duplications), suggesting genes encoded in this region may be the molecular link between Del3p and ASD. Some studies were unable to provide high-resolution analysis of chromosomal breakpoints, only providing general coordinates (e.g., 3p26 rather than 3p26.1, 3p26.2, or 3p26.3). Additionally, cases of chromosomal translocations involving chromosome 3p have been included within the “Deletion” category for ease of visualization. In cases of inherited CNVs, if the phenotype of the parent is discussed, they have also been included as well. Figure created using GraphPad Prism 8.

To identify which cytoband of chromosome 3p holds the largest burden for CNVs, the collated CNVs were mapped along the chromosome by the cytoband of their distal breakpoint (Figure 3). A significant portion of these CNVs (approximately 41.9%) are observed to occur in the 3p26.3 region, suggesting that mutations in genes encoded in this cytoband are important contributors to the neurodevelopmental phenotypes observed in individuals affected by Del3p. However, why is there such a high frequency of CNVs occurring at the 3p26.3 region? Although outside the scope of this review, the high frequency of CNVs in the 3p26.3 region could be explained by telomere attrition. Studies have previously reported higher frequency of ASD cases in individuals with shorter telomere lengths (Li et al., 2014), and that families at high-risk for ASD possess significantly shorter telomere lengths (Nelson et al., 2015). In spite of this, the high frequency of CNVs in the 3p26.3 region identifies this cytoband as a potential genetic risk locus for the ASD and ASD-associated phenotypes observed in individuals affected by Del3p.

It is important to acknowledge that just as ASDs present with a complex range of phenotypes (ranging from mild to severe), individuals affected by CNVs in chromosome 3p demonstrate a similar spectrum of phenotypic severity. Although the 3p26.3 region represents a promising candidate region for the neurodevelopmental phenotypes of Del3p, CNVs in the 3p26.3 region demonstrate incomplete penetrance for ASD and other neurodevelopmental phenotypes (Pohjola et al., 2010; Moghadasi et al., 2014). This suggests that depending on the location and magnitude of CNVs in chromosome 3p26.3, deletion or duplication of specific regions within the *CHL1*, *CNTN6*, and *CNTN4* genes may confer greater risk toward ASD development, and that these genes function as genetic modifiers to affect the severity of neurodevelopmental phenotypes. Interestingly, larger CNVs that encompass more genes appear to show higher penetrance and tend to result in more severe phenotypes. Guo et al. (2012) reported two cases of ASD with duplications affecting both *CNTN6* and *CNTN4*, which demonstrated striking deficits in social interaction and communication, in addition to markedly delayed development, seizures, speech delay and self-injurious behaviors. Other studies have also reported cases of CNVs affecting multiple neuronal IgCAMs in the 3p26.3 region in individuals diagnosed with ASD or those exhibiting pronounced autistic features (Schinzel, 1981; Drumheller et al., 1996; Angeloni et al., 1999; Fernandez et al., 2004; Malmgren et al., 2007; Pinto et al., 2010; van Daalen et al., 2011; Moghadasi et al., 2014; Weehi et al., 2014). These reports provide further evidence that mutations in the neuronal IgCAMs encoded at the 3p26.3 region produce additive deleterious effects that contribute to more severe phenotypes.

### Other Candidate Genes for Neurodevelopmental Phenotypes in Chromosome 3p

Although it is outside the scope of this review, other genes in chromosome 3p may also contribute to phenotypes observed in Del3p. Next to *CNTN4* in 3p26.2, three genes are present that are often lost in larger terminal 3p26.3 deletions: *Interleukin 5 Receptor Subunit Alpha* (*IL5RA*), *Transfer RNA Nucleotidyl Transferase 1* (*TRNT1*), and *Cereblon* (*CRBN*). *CRBN* in particular has been associated with autosomal recessive non-syndromic cognitive disability (Bavley et al., 2018), and disruptions to this gene by CNVs may contribute to the cognitive impairments observed in individuals affected by Del3p. Additionally, interstitial CNVs involving more proximal cytobands of the p-arm have also been associated with ASD (Harvard et al., 2005). CNVs affecting genes in these proximal cytobands have also been reported in individuals with ASD, PDD-NOS, language deficits, epilepsy, anxiety, and intellectual disability (Bittel et al., 2006; Ţuţulan-Cuniţă et al., 2012; Lesca et al., 2012; Riess et al., 2012; Schwaibold et al., 2013; Kellogg et al., 2013; Palumbo et al., 2013; Okumura et al., 2014; de la Hoz et al., 2015; Kuechler et al., 2015; Liu et al., 2015; Parmeggiani et al., 2018). A few genes encoded in chromosome 3p should be noted in relation to the neuronal IgCAMs at the 3p26.3 region – namely *Protein Tyrosine Receptor Phosphatase (PTPR) Type G* (*PTPRG*), *Fez Family Zinc Finger Protein 2* (*FEZF2*), and *Contactin-3* (*CNTN3*). *CNTN3* is encoded in the 3p12.3 region and belongs to the same subfamily of proteins as CNTN6 and CNTN4 and similarly, functions as a neuronal IgCAM to promote neurite outgrowth (Yoshihara et al., 1995). PTPRG is a protein tyrosine phosphatase that has previously been found to interact with CNTN3, CNTN4, and CNTN6 (Bouyain and Watkins, 2010a,b), and this interaction regulates neurite outgrowth (elaborated upon later in section “Axon Guidance, Neurite Outgrowth and Neuronal Migration” of this review) (Mercati et al., 2013). Furthermore, Parmeggiani et al. (2018) recently showed through a STRING protein interaction network analysis that FEZF2 interacts indirectly with PTPRG via CNTN4, placing these proteins in the same interactome network. This may explain why both interstitial and terminal CNVs in chromosome 3p result in similar phenotypes, as the affected genes participate in convergent neurodevelopmental pathways.

## Neurophysiological Roles of *CHL1, CNTN6*, and *CNTN4*

### Spatiotemporal Expression Patterns of CHL1, CNTN6, and CNTN4

**FIGURE 4:**
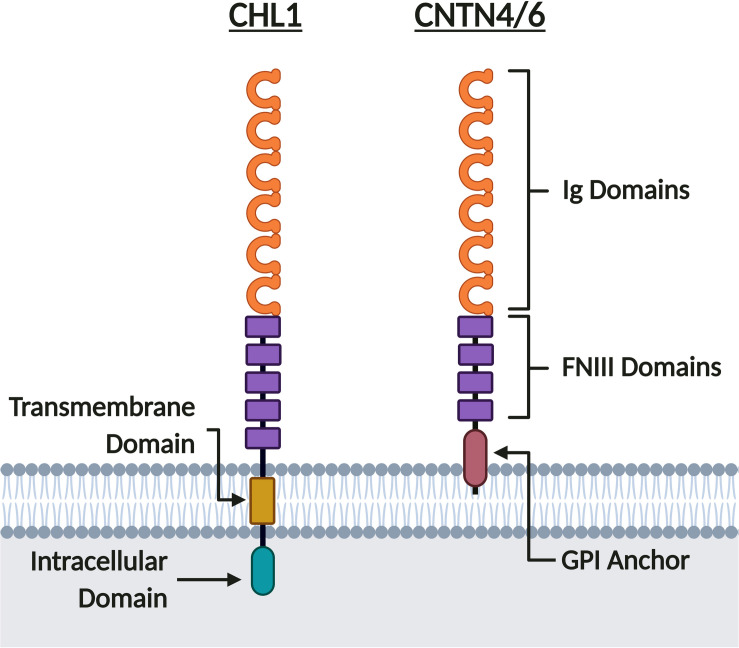
CHL1, CNTN6, and CNTN4 share similar protein structure. CHL1, CNTN6, and CNTN4 are members of the IgCAM superfamily. These neuronal IgCAMs share similar extracellular domains composed of six Ig domains and five FNIII domains for CHL1, or four FNIII domains for CNTN6 and CNTN4. Whereas CNTN6 and CNTN4 are tethered to the membrane by a GPI anchor, CHL1 has both transmembrane and intracellular domains. Created with BioRender.com.

The three neuronal IgCAMs encoded in the 3p26.3 region belong to the Immunoglobulin (Ig) CAM (IgCAM) superfamily (Holm et al., 1996; Fusaoka et al., 2006; Shimoda and Watanabe, 2009; Chatterjee et al., 2019). Within the IgCAM superfamily, CHL1 belongs to the L1 family, whilst CNTN6 and CNTN4 belong to the Contactin family (Figure 4). As such, they share a common extracellular domain comprised of six N-terminal Ig domains followed by five Fibronectin Type III (FNIII) domains for CHL1 (Holm et al., 1996), or four FNIII domains for CNTN6 and CNTN4 (Shimoda and Watanabe, 2009; Figure 4). CHL1 contains a transmembrane and intracellular C-terminal domain, however CNTN6 and CNTN4 lack an intracellular domain and are instead tethered to the cell membrane by a C-terminal glycosylphosphatidylinositol (GPI) anchor (Holm et al., 1996; Maness and Schachner, 2007; Shimoda and Watanabe, 2009; Figure 4). As CNTN6 and CNTN4 do not have intracellular domains, they require interacting partners for intracellular signal transduction or act directly as ligands for receptors on opposing cells. Due to their structural similarity, it is likely that these neuronal IgCAMs interact with similar extracellular ligands during neurodevelopment (elaborated upon in sections “Axon Guidance, Neurite Outgrowth, and Neuronal Migration,” “Synaptic Transmission and Plasticity,” and “Behavior Phenotypes of Animal Models”). These neuronal IgCAMs also share some overlapping but distinct expression patterns, suggesting that they contribute to a combinatorial code (Yamagata et al., 2002). Previous studies within the rodent forebrain and cortex indicate that the temporal expression of all three neuronal IgCAMs follows a similar pattern – starting low during the final week of gestation and rising to peak expression levels during the first week of postnatal development (Yoshihara et al., 1995; Hillenbrand et al., 1999; Lee et al., 2000; Kaneko-Goto et al., 2008; Oguro-Ando et al., 2017). Post gestation, the expression of Chl1 and Cntn6 falls to a constant level (Hillenbrand et al., 1999; Lee et al., 2000), whereas the expression of Cntn4 continues to increase until a constant level that is maintained in adulthood (Yoshihara et al., 1995; Kaneko-Goto et al., 2008).

Cell-type-specific expression of these neuronal IgCAMs throughout neurodevelopment, from previously reported data in rodent models, is not so straightforward. Chl1 is reported to be strongly expressed in cortical layer V interneurons, hippocampal pyramidal cells of the Cornu Ammonis 1 and 3 (CA1 and CA3, respectively) regions, and in interneurons of the hippocampus proper and the hilar region of the dentate gyrus (DG) (Hillenbrand et al., 1999). Cntn6 is significantly expressed in the cortex within layer V interneurons, and within the CA1 hippocampus region, the DG and the hilar region of the DG (Lee et al., 2000; Zuko et al., 2016a,b). Cntn4 is strongly expressed within layer V cortical interneurons, and although weakly expressed in regions of the hippocampus, is specifically localized to granule cells of the DG (Chatterjee et al., 2019). Although Chl1, Cntn6, and Cntn4 are all expressed in layer V interneurons of the cortex, Cntn6 is more prominently expressed within layers Vb and VIb whereas Cntn4 is more strongly expressed in layers Vb and VIa (Oguro-Ando et al., 2017). Furthermore, while Cntn4 is extensively expressed in layers II–V of the cortex, Cntn6 expression is more confined to layers II, III, and V (Zuko et al., 2016b; Oguro-Ando et al., 2017). Although our knowledge of cell-type-specific expression of these neuronal IgCAMs is limited, these studies in rodent models suggest that Chl1, Cntn6, and Cntn4 display overlapping, though distinct, spatiotemporal expression patterns. Advances in single-cell transcriptomic profiling technology have provided greater insight into cell-type-specific expression patterns. For example, the *Allen Brain Atlas* has curated single-cell and single-nucleus RNA-sequencing data to produce a transcriptional profile of different regions of the human and mouse cortex and hippocampus (©2015 Allen Institute for Brain Science. Allen Cell Types Database. Available from https://celltypes.brain-map.org/). Single-cell transcriptomic datasets such as these are vital to improving our understanding of CHL1, CNTN6, and CNTN4 expression in the developing human brain.

**FIGURE 5:**
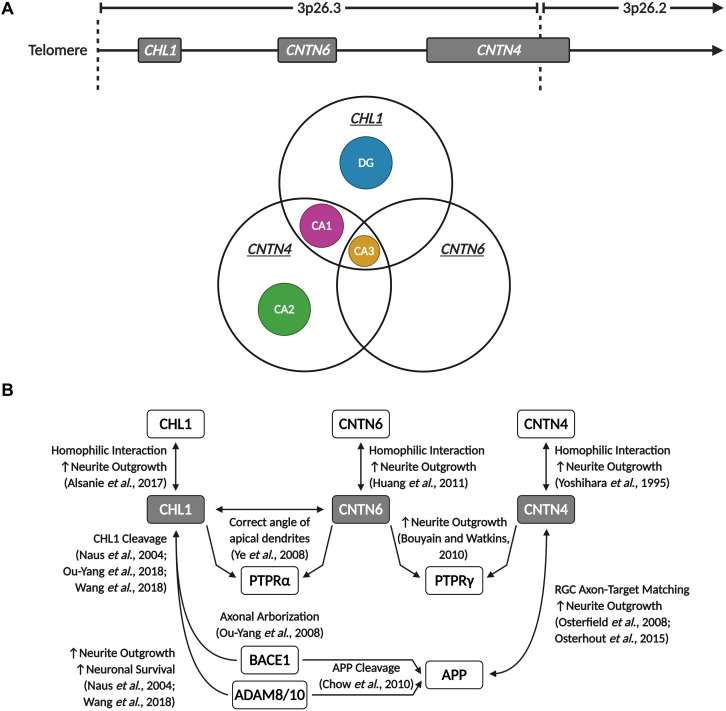
*CHL1*, *CNTN6*, and *CNTN4* display overlapping expression in the hippocampus and participate in shared signaling pathways. **(A)** *Chl1*, *Cntn6*, and *Cntn4* demonstrate region-specific and overlapping expression patterns in the mouse hippocampus. The CA2 and DG only express *Cntn4* and *Chl1*, respectively, while all three neuronal IgCAMs are co-expressed in the CA3, and *Chl1* and *Cntn4* are co-expressed in the CA1. Data derived from the *Allen Brain Atlas* (©2015 Allen Institute for Brain Science. Allen Cell Types Database. Available from https://celltypes.brain-map.org/). **(B)** CHL1, CNTN6, and CNTN4 are capable of homophilic interactions that promote neurite outgrowth. These three neuronal IgCAMs commonly interact with members of the PTPR family – CHL1 interacts with CNTN6 to direct and maintain the correct angle of apical dendrites in cortical pyramidal neurons through PTPRα signaling, and both CNTN6 and CNTN4 are reported to interact with PTPRγ to promote neurite outgrowth. Additionally, CHL1 and CNTN4 interact with APP and enzymes involved in the proteolytic processing of APP – CNTN4 interacts with APP to promote neurite outgrowth and regulate retinal ganglion cell (RGC) axon-target matching in the development of visual pathways, and CHL1 cleavage by BACE1 and ADAM proteins is observed to promote neuronal survival, neurite outgrowth and regulate axonal arborization. Created with BioRender.com.

In the human motor cortex, *CHL1* and *CNTN4* are, with a few exceptions, commonly expressed in most GABAergic interneuron and glutamatergic pyramidal neuron populations (©2015 Allen Institute for Brain Science). However, *CNTN6* demonstrates striking cell-type-specific expression, mainly being expressed in Vasoactive Intestinal Peptide (VIP), Somatostatin (SST), and Parvalbumin (PVALB) expressing interneurons, and Thymocyte Selection Associated (THEMIS), RAR-Related Orphan Receptor B (RORB) and FEZF2 expressing pyramidal neurons (©2015 Allen Institute for Brain Science). In the mouse hippocampus, the expression of these neuronal IgCAMs becomes more selective. Whilst *Cntn4* is expressed in the CA1, CA2, and CA3 regions, *Chl1* is only expressed in the CA1 and CA3, and *Cntn6* is only expressed in the CA3 (Figure 5A). In the DG, low levels of *Cntn6* and *Cntn4* can be detected, whilst *Chl1* is expressed in granule cells and GABAergic interneuron populations (Hochgerner et al., 2018; Figure 5A). This demonstrates that each of these neuronal IgCAMs has its own expression pattern independent from one other. The overlapping expression indicates that neurons can differ in combinations of *CHL1*, *CNTN6*, and *CNTN4* expression – these combinatorial patterns [suggest] that these IgCAMs are part of a code, as has been suggested for Contactins in the retina (Yamagata and Sanes, 2012).

Regardless of their individual expression patterns, the common peak temporal expression during early postnatal development suggests that CHL1, CNTN6, and CNTN4 play key roles in synaptogenesis and the maintenance of neuronal networks within the developing brain. Their similar structure and partially overlapping spatial expression indicates that disruptions to the expression or function of these neuronal IgCAMs could produce additive deleterious effects. To understand the co-morbidities of these proteins in the brain, further investigations and confirmation of the developmental expression of *CHL1*, *CNTN6*, and *CNTN4* expression in the human brain is essential. In subsequent sections of this review, we will further discuss these neuronal IgCAMs and their functional role in neurons, including common interacting partners. In the case of overlapping interactomes, we will study their contribution toward ASD etiology.

### Regulation of Neurogenesis and Neuronal Survival

Dysregulated neurogenesis and neuronal apoptosis are phenotypes that are associated with ASD – both in animal models and postmortem brains (Wegiel et al., 2010; Wei et al., 2014; Fan and Pang, 2017; Courchesne et al., 2019). The neuronal IgCAMs encoded in the 3p26.3 region have demonstrated roles in promoting neuronal survival and regulating neurogenesis. Chl1 has been observed to suppress apoptosis in primary cultures (Chen et al., 1999; Naus et al., 2004; Nishimune et al., 2005). Overexpression of Chl1 or its treatment in soluble or substrate form is hypothesized to exert anti-apoptotic effects by induction of Phosphatidylinositol-3-Kinase (PI3K) and Mitogen-Activated Protein Kinase (MAPK) signaling (Nishimune et al., 2005) and by increasing the expression of B-Cell Lymphoma 2, an anti-apoptotic protein (Chen et al., 1999). Notably, altered PI3K and MAPK signaling is implicated in ASD and other neurodevelopmental disorders (Enriquez-Barreto and Morales, 2016; Baranova et al., 2020). Chl1 has also been observed to interact with the Patched-1 hedgehog receptor in regulation of neuronal survival, as inhibitors of Smoothened and RhoA and Rho-Associated Kinases 1 and 2 are able to inhibit Chl1-mediated survival of cerebellar Purkinje and granule cells (Katic et al., 2017). Studies in Chl1-deficient mice have also revealed increased apoptosis, loss of PVALB-positive (PV+) neurons and decreased precursor cell proliferation in the CA1 region of the hippocampus and cerebellum (Jakovcevski et al., 2009; Schmalbach et al., 2015). Interestingly, the loss of PV+ interneurons in the hippocampus is correlated with increased microglial activation and enhanced IL-6 secretion in the hippocampus (Schmalbach et al., 2015). Although it is outside the scope of this review, dysregulated neurogenesis as a result of neuroinflammation has also been observed in ASD (Fan and Pang, 2017), suggesting a mechanism exists by which Chl1 and other neuronal IgCAMs may regulate inflammatory signaling in the brain.

Although there are limited studies, Cntn6 and Cntn4 are also reported to play roles in regulating neuronal survival. Cntn6 regulates neuronal survival and morphology through its interaction with Latrophilin-1 (Lphn1) (Zuko et al., 2016a). Neurons overexpressing Lphn1 display morphological defects including reduced neurite branching points, soma size, total neurite length, and longest branch length (Zuko et al., 2016a). Increased Caspase-3 immunoreactivity was also observed in the Cntn6-deficient visual cortex (Zuko et al., 2016a). Interestingly, Lphn1 is known to interact with Neurexin-1, another neuronal IgCAM whose disruption is also associated with ASD (Kim et al., 2008; Moreno-Salinas et al., 2019). These interactions between Lphn1, Cntn6, and other neuronal IgCAMs may point toward a common cellular pathway implicated in ASD etiology.

### Axon Guidance, Neurite Outgrowth, and Neuronal Migration

Deficits in neuronal migration and axon guidance are a characteristic commonly observed in ASD (Wegiel et al., 2010; Pan et al., 2019). In Chl1-deficient mice, impairments in neuritogenesis and axonal guidance can be observed. In the hippocampus, mossy fibers of the CA3 region display unorganized projections that invade into the pyramidal cell layer, rather than forming the supra- and infra-pyramidal bundles parallel to the pyramidal cell layer observed in wild-type mice (Montag-Sallaz et al., 2002; Frints et al., 2003; Heyden et al., 2008). Additionally, Chl1-deficient mice display aberrant arborization of olfactory sensory neuron axons (Montag-Sallaz et al., 2002; Heyden et al., 2008). Chl1 is further reported to stimulate neurite outgrowth in primary neuronal cultures (Chen et al., 1999; Hillenbrand et al., 1999; Dong et al., 2002), and may promote neurite outgrowth via multiple pathways. For example, the intracellular domain of Chl1 interacts with Ezrin, a member of the Ezrin-Radixin-Moesin (ERM) family of Actin-binding proteins, to modulate Actin dynamics and facilitate F-Actin remodeling (Schlatter et al., 2008). The Chl1-Ezrin interaction also plays a role in axonal repulsion by mediating the growth cone collapsing activity of the chemorepellent Semaphorin-3A (Sema3a) (Schlatter et al., 2008), and insufficient Sema3a-mediated repulsion in CHL1-deficient mice is observed to alter the positioning of axons projecting toward the cortex from the ventral telencephalon and basal complex (Wright et al., 2007). Chl1 binding to Sema3a triggers Chl1 cleavage by β-Site Amyloid-Precursor Protein-Cleaving Enzyme 1 (Bace1), which generates an active membrane-bound Chl1 fragment that interacts with Ezrin to relay the Sema3a signal to the Actin cytoskeleton (Barão et al., 2015). In addition to Sema3a, Chl1 also interacts with other molecules in the growth cone to regulate axon targeting and neurite outgrowth, including the Ephrin A7 Receptor (Demyanenko et al., 2011) and Disrupted in Schizophrenia 1 (Ren et al., 2016). Chl1 may also promote neuronal migration and neurite outgrowth by interacting with β1 Integrins and recruiting the Actin-binding protein Ankyrin to the cell membrane, triggering activation of c-Src, PI3K, and MAPK cascades (Buhusi et al., 2003; Demyanenko et al., 2004; Katic et al., 2014). More recently, homophilic Chl1–Chl1 interactions have also been observed to regulate neurite outgrowth during the development of ventral midbrain dopaminergic pathways (Alsanie et al., 2017).

Interestingly, Chl1 was observed to interact with Cntn6, and this interaction is responsible for maintaining the correct angle of apical dendrites in cortical pyramidal neurons through Ptpr-Alpha (Ptpra) signaling (Ye et al., 2008). Notably, compound heterozygous mice for both Chl1 and Cntn6 display an additive deleterious phenotype – these compound heterozygous mice showed more severe misoriented dendrites compared to single heterozygous Chl1 or Cntn6 mice and wild-type littermates (Ye et al., 2008). This suggests that Chl1 and Cntn6 may partially compensate for one another, and places Cntn6 directly within Chl1’s interactome. Although no studies have validated this further, it is possible Cntn6 could interact with the same binding partners as Chl1 as well, thereby participating in shared intracellular pathways (Zuko et al., 2013). It is worthwhile to note that *Chl1* and *Cntn6* are both expressed in the CA3 region of the hippocampus (Figure 5A), and that Cntn6-deficient mice also display a larger suprapyramidal bundle (Zuko et al., 2016b), a phenotype similar to that observed in the hippocampus of Chl1-deficient mice (Montag-Sallaz et al., 2002; Frints et al., 2003; Heyden et al., 2008). In addition to its interaction with Chl1, Cntn6 is also reported to regulate neurite outgrowth in primary neuronal cultures through Ptprg signaling (Takeda et al., 2003; Bouyain and Watkins, 2010b; Mercati et al., 2013). Importantly, Cntn4 has been observed to interact with Ptprg, and this interaction promotes neurite extension (Bouyain and Watkins, 2010b; Cottrell et al., 2011). Therefore, at least within the context of neuritogenesis, these three neuronal IgCAMs share an overlapping interactome, as they interact with each other and with similar binding partners (Figure 5B). This suggests that these neuronal IgCAMs participate in common neurodevelopmental pathways and may display some functional redundancy, leading to additive deleterious phenotypes with loss-of-function mutations. Additionally, similar to Chl1, Cntn6 is capable of promoting neurite outgrowth through homophilic Cntn6–Cntn6 interactions (Huang et al., 2011). As well as promoting neuritogenesis, Cntn6 is also observed to play roles in axon guidance, particularly in the establishment of the corticospinal tract (Huang et al., 2012). Cntn6-deficient mice display a delay in the development of projections of corticospinal tract axons during prenatal and neonatal development (Huang et al., 2012).

Contactin-4 is observed to play roles in regulating axon guidance and neurite extension, for example promoting neurite outgrowth in primary cortical neurons (Yoshihara et al., 1995; Mercati et al., 2013). Similar to Cntn6, Cntn4 may interact with Chl1 or other members of the L1 family, and both heterophilic and homophilic Cntn4 interactions may promote neurite extension (Yoshihara et al., 1995; Mercati et al., 2013). Cntn4’s role in axon guidance has been extensively explored in multiple species in the development of olfactory and visual pathways (Kaneko-Goto et al., 2008; Osterfield et al., 2008). In the olfactory system, Cntn4 expression in axon terminals guides individual axons from odorant receptors to distinct olfactory bulb glomeruli, suggesting Cntn4 promotes the formation and maintenance of odor maps, possibly via interaction with Ephrin-A5 (Kaneko-Goto et al., 2008). Similar to Chl1, Cntn4-deficient olfactory sensory neurons display aberrant projections toward multiple olfactory bulb glomeruli (Kaneko-Goto et al., 2008). This shared axon guidance deficit observed between Chl1- and Cntn4-deficient mice further supports the possibility that these neuronal IgCAMs participate in common signaling pathways during neurodevelopment. Osterfield et al. (2008) identified extracellular binding partners of Amyloid Precursor Protein (App) in retinal axons growing on the optical tectum, which is a well characterized model of axon development (McLaughlin and O’Leary, 2005; Flanagan, 2006). In retinal ganglion cells, Cntn4 is suitably placed to interact with App during the developing retinotectal system in order to mediate axon outgrowth and axon-target matching. Cntn4-deficiency results in defects in retinal ganglion cell axon-target matching, arborization and outgrowth (Osterfield et al., 2008; Osterhout et al., 2015). This system is clearly sensitive to axon guidance defects or to absence of certain CAMs, as was demonstrated in Bace1-knockout mice (Hitt et al., 2012). Although axon guidance deficits in the hippocampus of Cntn4-deficient mice have not yet been explored, it would be interesting to see if Cntn4-deficient mice also display abnormal mossy fiber projections similar to Chl1- and Cntn6-deficient mice, as *Cntn4* is expressed in the CA3 region as well (Figure 5A).

Interestingly, cleavage of these neuronal IgCAMs by specific enzymes may also be key to their functions in axon guidance and promoting neurite outgrowth. For example, Chl1 cleavage by Bace1 has been demonstrated to be involved in axonal organization, as reduced Bace1-mediated Chl1 cleavage is reported to contribute toward defective axonal organization in Bace1-deficient mice (Ou-Yang et al., 2018). Notably, Bace1-deficient mice display axon guidance defects in the olfactory bulb and hippocampus similar to Chl1 and Cntn4-deficient mice (Hitt et al., 2012). BACE1, together with proteins of the Disintegrin and Metalloproteinase Domain-Containing Protein (ADAM) family, are key enzymes in APP processing (Sinha et al., 1999; Vassar et al., 1999). APP cleavage by ADAM family proteins (α-secretase activity) or BACE1 (β-secretase activity) produces a secreted APP (sAPP) fragment and a membrane-bound carboxyl terminal fragment (CTF) – these fragments are referred to as sAPPα/β and CTFα/β depending on their generation via α- or β-secretase activity (Chow et al., 2010). In addition to its interaction with APP, CNTN4 may also play a modulatory role in APP processing, as co-expression of CNTN4 with APP in transfected HEK-293T cells resulted in an increase production of CTFα (Osterfield et al., 2008). It is unknown if CNTN4 also regulates the activity of or is cleaved by ADAM proteins, but these findings suggest that CHL1 and CNTN4 may play similar roles in an overlapping pathway regulating APP processing. Other studies have also reported that Adam8 cleaves Chl1 to release an extracellular fragment that promotes neuronal survival and neurite outgrowth *in vitro* (Naus et al., 2004), and Adam10 interacts with Bace1 to regulate Chl1 cleavage (Wang et al., 2018). The Adam10-Bace1 interaction may be important for neuritogenesis in primary neuronal cultures and importantly, APP is reported to promote neuronal migration and neurite outgrowth (Billnitzer et al., 2013; Nicolas and Hassan, 2014; Wang et al., 2017). Increased levels of sAPPα are also associated with ASD, hinting toward dysregulated APP processing as a convergent pathway in ASD etiology (Ray et al., 2011; Sokol et al., 2019).

### Synaptic Transmission and Plasticity

Neuronal IgCAMs have been found to influence synapse formation, maintenance and plasticity (Betancur et al., 2009; Sytnyk et al., 2017). L1 and Contactin family neuronal IgCAMs perform important functions at neuronal synapses, forming homodimer and heterodimer *trans* complexes with other neuronal IgCAMs or receptors. As discussed in section “Axon Guidance, Neurite Outgrowth, and Neuronal Migration,” these neuronal IgCAM interactions are important for axon guidance, target-matching, and arborization, but they also anchor, organize, and bridge the synaptic cleft (Missler et al., 2012; Yang et al., 2014; Chatterjee et al., 2019). In this section we focus on the reported non-adhesion functions of CHL1, CNTN6, and CNTN4 in synaptic transmission and how they may influence synaptic plasticity.

In addition to promoting the formation and stabilization of synapses (Ashrafi et al., 2014), CHL1 is reported to play multiple non-adhesion roles that modulate synaptic activity. The intracellular domain of Chl1 regulates synaptic vesicle recycling by interacting with the 70 kDa Heat Shock Cognate (Hsc70) synaptic chaperone protein and regulating the un-coating of Clathrin-coated synaptic vesicles in the Clathrin-dependent recycling pathway (Leshchyns’ka et al., 2006). Chl1 localizes primarily in the presynaptic terminals of axons of both excitatory and inhibitory neurons, co-localizing with Hsc70 and the synaptic vesicle marker Sv2 (Leshchyns’ka et al., 2006). Chl1 also modulates the refolding of Soluble N-Ethylmaleimide-Sensitive Factor Attachment Protein Receptor (SNARE) complex proteins to maintain the vesicle recycling machinery during periods of continuous synaptic activity (Andreyeva et al., 2010). Interestingly, Chl1 was recently reported to interact with Dopamine Receptor D2 (DRD2), regulating its internalization at the presynaptic environment (Kotarska et al., 2020). This suggests that Chl1 may regulate dopaminergic signaling in neurons, and also the density of neurotransmitter receptors on the surface of axon terminals, which can influence how the presynaptic environment responds to modulatory signals from axo-axonic synapses. Chl1 has also been observed to play roles in Serotonin-2C Receptor (5-ht2cr) signaling in hippocampal GABAergic neurons – its deficiency impairs 5-ht2cr phosphorylation and 5-ht2cr association with Phosphatase and Tensin Homolog (Pten) and Arrestin Beta-2 (Arrb2) (Kleene et al., 2015). Notably, *PTEN* and *ARRB2* are both implicated in ASD etiology as well (Varga et al., 2009; Thompson and Dulawa, 2019). Genetic variants in *CHL1*, *ITGB1*, and *ITGB3* have also been identified as predictors of treatment-resistant depression and responsiveness to selective serotonin reuptake inhibitors (SSRIs), suggesting that the Chl1-Integrin interactions that regulate neuronal migration have further roles in serotonergic signaling (Morag et al., 2011; Oved et al., 2013, 2017; Fabbri et al., 2015, 2017; Probst-Schendzielorz et al., 2015). Several studies have also observed long-term potentiation (LTP) deficits at synapses between the CA3 and CA1 region of the hippocampus that could be rescued by application of GABA_A_R modulators, indicating that Chl1 may play a role in the establishment of synaptic plasticity as well (Nikonenko et al., 2006; Schmalbach et al., 2015). Taken altogether, these studies demonstrate that CHL1 and its interactors regulate a complex array of synaptic functions, ranging from synaptic vesicle recycling to neurotransmitter receptor internalization and downstream signaling.

As Chl1 has been observed to interact with Cntn6 within cortical neurons (Ye et al., 2008), and both neuronal IgCAMs share overlapping expression patterns in certain brain regions and neuronal populations, it is also possible Cntn6 may participate in similar functions to Chl1 in the presynaptic environment. Although there have been no direct studies investigating the role of Cntn6 in vesicular recycling, Cntn6 is expressed in the presynaptic terminals of glutamatergic synapses in the hippocampus where it interacts with Vglut1 and Vglut2, transporters that regulate glutamate uptake into presynaptic vesicles (Sakurai et al., 2009, 2010). Cntn6-deficiency is observed to reduce Vglut1 and Vglut2 expression in the hippocampal formation, suggesting that Cntn6 participates in the formation and maintenance of glutamatergic synapses (Sakurai et al., 2010). These studies point toward Cntn6’s involvement in a shared pathway regulating vesicle processing at the presynapse with Chl1. Similar to the regulation of apical dendrites in pyramidal neurons (Ye et al., 2008), this could be another mechanism by which Cntn6 can compensate for Chl1 deficiency, and future studies should investigate if Cntn6 regulates vesicular recycling in a similar manner to Chl1. Interestingly, Cntn6-deficiency is also observed to cause a decrease in the number of PV+ GABAergic interneurons in the visual cortex (Zuko et al., 2016a). These studies indicate that Cntn6 may be a regulator of synaptic transmission in both excitatory and inhibitory neuron populations within the brain. It would therefore be interesting investigate if the effects of Cntn6 deficiency in cortical and hippocampal neurons translates to a shift in excitation-inhibition (E/I) ratios and changes in synaptic plasticity, as increases in E/I ratios due to reduced GABAergic signaling have been associated with deficits in social interaction and other neuropsychiatric phenotypes (Yizhar et al., 2011).

A recent study observed that Cntn4-deficient mice display altered cell-surface expression of glutamate and GABA receptors (GABARs) in ASD-related brain regions (Heise et al., 2018). Cntn4-deficient mice show reduced cell-surface glutamate and GABA_A_Rα1 receptor levels in the cerebral cortex and hippocampus, suggesting Cntn4 can modulate synaptic transmission by altering the density of cell-surface neurotransmitter receptors (Heise et al., 2018). However, whether Cntn4 deficiency impairs glutamatergic or GABAergic signaling within cortical and hippocampal neurons, and subsequently whether these changes in cell-surface receptor expression result in altered E/I ratios or changes in LTP remains to be fully determined. *Cntn4* is expressed in both the CA1 and CA3 regions (Figure 5A), so it would be interesting to see if Cntn4-deficient mice display decreased PV+ neurons in the CA1 region and LTP deficits at CA3-CA1 synapses, similar to Chl1-deficient mice (Nikonenko et al., 2006; Schmalbach et al., 2015). Additionally, due to its high structural similarity with Cntn6, and shared binding partners with Cntn6, it is likely Cntn4 may perform similar functions to Cntn6 or even Chl1 at neuronal synapses. Therefore, it is possible these neuronal IgCAMs can compensate for each other’s loss of function, and that disruptions to multiple genes can produce additive deleterious phenotypes.

Members of the Contactin-Associated Protein (CASPR) family interact and modulate the activity of Contactins (Murai et al., 2002; Gollan et al., 2003; Ashrafi et al., 2014; Rubio-Marrero et al., 2016). Importantly, several CASPR proteins have been reported to regulate the function of neurotransmitter receptors. CASPR1 and CASPR2 are observed to disrupt α-Amino-3-Hydroxy-5-Methyl-4-Isoxazolepropionic Acid receptor (AMPAR) function, causing synaptic abnormalities and regulating trafficking to the synapse (Santos et al., 2012; Fernandes et al., 2019; Gao et al., 2019). This presents a hypothesis that neuronal IgCAMs such as CNTN6 and CNTN4 could form complexes whose interactions regulate AMPAR and GABAR function and synaptic plasticity. Heise et al. (2018) suggests that CNTN4 may act as a molecular chaperone for cell surface expression of neurotransmitter receptors. It is worthwhile to note that CNTN6 has been observed to perform a similar role as well (Ye et al., 2011). Considering that Contactins lack intracellular signaling domains, it is likely that in certain cells CNTN6 and CNTN4 act as part of a larger complex of proteins, serving as chaperones or as ligands to initiate and inhibit molecular pathways. A similar situation may be the case between CHL1 and BACE1 (Hitt et al., 2012; Ou-Yang et al., 2018). Taking into account that CHL1 and CNTN6 interact with each other, the formation of a protein complex comprised of CHL1, CNTN6, and various CASPR proteins could regulate a range of synaptic functions. Ashrafi et al. (2014) demonstrated an example of such a complex in the mouse spinal cord, in which a Chl1-Nrcam heterodimer interacts in *trans* with a Cntn5-Caspr4 heterodimer to promote the formation of and stabilize axo-axonic synapses. These collective interactions merit further investigation to decipher the heterodimeric complexes and signaling pathways CHL1, CNTN6, and CNTN4 are involved in at the synapse.

### Behavior Phenotypes of Animal Models

The use of validated neurobehavioral tests for rodents provides an avenue for researchers to understand how specific genetic alterations impact molecular pathways that alter neuronal development and synaptogenesis. Understanding how these alterations affect brain connectivity and function is essential to improve our understanding of the core behavioral features of ASD (Zerbi et al., 2018). To model the behavior phenotypes of Del3p, it important to note that the 3p26.3 cytoband encoding *CHL1*-*CNTN6*-*CNTN4* is syntenic to the mouse chromosome 6qE1. Several studies have extensively characterized the phenotype of Chl1-deficient mice (Table 2). The use of SHIRPA behavior test batteries (Rogers et al., 1997, 2001) has revealed that Chl1-deficient mice display altered exploratory behavior, reduced behavior flexibility and novelty detection, impaired motor coordination, and altered social interaction and spatial information processing. Chl1-deficient mice are consistently observed to exhibit altered exploratory behavior and reduced anxiety in tests such as the open-field test, elevated-plus maze, light-dark transition test, and the Morris water maze (Montag-Sallaz et al., 2002; Frints et al., 2003; Pratte et al., 2003; Pratte and Jamon, 2009). This altered exploratory behavior may be due to impaired spatial and object novelty detection (Pratte et al., 2003; Morellini et al., 2007; Pratte and Jamon, 2009). Furthermore, Chl1-deficient mice are observed to have deficits in motor coordination, struggling to remain balanced in the rotarod test (Pratte et al., 2003). Chl1-deficient mice also display increased passivity and reduced aggressiveness, including reduced stress responses associated with transfer arousal, touch escape and irritability (Frints et al., 2003; Pratte et al., 2003; Morellini et al., 2007). Importantly, Chl1-deficient mice display altered social preference, increased behavioral inflexibility, and deficits in integrating spatial and temporal information in the hippocampus (Montag-Sallaz et al., 2002; Frints et al., 2003; Morellini et al., 2007; Buhusi et al., 2013). These are phenotypes that have been observed in certain mouse models for ASD such as the Fmr1-, Shank3-, Nlgn4-, Caspr2-, and Pten-deficient mice (Kwon et al., 2006; Jamain et al., 2008; Peñagarikano et al., 2011; Duffney et al., 2015; Kazdoba et al., 2016; Ricceri et al., 2016; Gurney et al., 2017; Verma et al., 2019).

**TABLE 2:** Behavior phenotypes observed in animal models for CHL1, CNTN6, and CNTN4 deficiency.

Mouse Model	Cognitive Domain	Task (If Applicable)	Phenotype	References
Chl1^–/–^ mouse	Aggressiveness	Resident-intruder test	Reduced	Frints et al., 2003; Morellini et al., 2007
			Reduced social urine marking behavior	Morellini et al., 2007
	Anxiety	Open-field test	Reduced	Montag-Sallaz et al., 2002
		Elevated-plus maze	Reduced	Montag-Sallaz et al., 2002
		Light/dark avoidance test	Unaffected	Montag-Sallaz et al., 2002; Pratte et al., 2003
	Behavioral or cognitive flexibility	Morris water maze	Reduced	Montag-Sallaz et al., 2002; Frints et al., 2003
	Exploratory behavior	Open-field test	Altered	Montag-Sallaz et al., 2002; Pratte et al., 2003; Pratte and Jamon, 2009
		Morris water maze	Altered	Montag-Sallaz et al., 2002; Frints et al., 2003
	Learning and memory	Radial-arm maze	Deficits in integrating spatial and temporal information	Buhusi et al., 2013
	Motor coordination	Rotarod test	Impaired	Pratte et al., 2003
	Novelty detection	Open-field test	Impaired	Pratte et al., 2003; Morellini et al., 2007; Pratte and Jamon, 2009
		Elevated-plus maze	Impaired	Morellini et al., 2007
		Novel object test	Impaired	Morellini et al., 2007
	Olfactory function		Unaffected	Morellini et al., 2007
	Social interaction	Social preference test	Delayed reactivity to initiate social investigation and preference for familiar animals	Morellini et al., 2007
	Stress response		Reduced stress associated with transfer arousal, touch escape, and irritability	Pratte et al., 2003
Cntn6^–/–^ mouse	Motor coordination	Rotarod test	Impaired	Takeda et al., 2003
	Learning and memory	Morris water maze	Impaired spatial learning	Mu et al., 2018
Cntn4^–/–^ mouse	Anxiety	Open-field test	Unaffected	Molenhuis et al., 2016
		Elevated-plus maze	Unaffected	Molenhuis et al., 2016
	Behavioral or cognitive flexibility	Barnes maze	Unaffected	Molenhuis et al., 2016
		Set-shifting task	Unaffected	Molenhuis et al., 2016
	Exploratory behavior	Open-field test	Unaffected	Molenhuis et al., 2016
		Elevated-plus maze	Unaffected	Molenhuis et al., 2016
	Learning and memory	Barnes maze	Enhanced spatial learning	Molenhuis et al., 2016
	Motor coordination	Rotarod test	Unaffected	Molenhuis et al., 2016
	Olfactory function	Buried food test	Unaffected	Molenhuis et al., 2016
	Repetitive behavior		No effect on time spent grooming	Molenhuis et al., 2016
	Responsiveness to stimuli		Increased startle response to auditory stimuli	Molenhuis et al., 2016
	Social interaction	Juvenile social interaction test	No effect on social sniffing, anogenital sniffing, or social grooming	Molenhuis et al., 2016
		Three-chamber social interaction test	No effect on social preference	Molenhuis et al., 2016
		Social discrimination test	No effect on social exploration or recognition	Molenhuis et al., 2016

In contrast to Chl1, the behavioral impacts of Cntn6 deficiency have not yet been well characterized (Table 2) and very few studies have examined the ASD-related behavioral effects of Cntn6 deficiency. Cntn6 deficiency was observed to result in deficits in spatial learning and memory in the Morris water maze task (Mu et al., 2018). In particular, male Cntn6-deficient mice exhibited slower spatial learning, which was attributed to altered hippocampal development (Mu et al., 2018). Another study investigated the effect of Cntn6 deficiency on motor coordination and observed that similar to Chl1-deficient mice, Cntn6-deficient mice display impaired motor coordination as well (Takeda et al., 2003). When subjected to the rotarod and horizontal rod tests, Cntn6-deficient mice struggled to stay and walk on the rods, indicating impaired balance control, despite muscle strength being unaffected (Takeda et al., 2003). Takeda et al. (2003) hypothesized that these defects in motor coordination were due to Cntn6 deficiency in the cerebellum, which leads to neuronal dysfunction. Notably, other studies have also reported that Chl1 plays an important role in cerebellar development, being expressed on the Bergmann glial cells that guide neuronal migration and synaptogenesis (Ango et al., 2008), and Chl1-deficient mice display increased neuronal apoptosis in the cerebellum (Jakovcevski et al., 2009). Importantly, cerebellar dysfunction has been previously associated with ASD (Wang et al., 2014; Hampson and Blatt, 2015; Mosconi et al., 2015). Zuko et al. (2016a) also hypothesized that as Cntn6-deficient mice display reduced numbers of PV+ GABAergic interneurons in the visual cortex, this shift in interneuron populations could lead to changes in E/I ratios in the cortex and affect behavior. Future studies into the behavioral impact of Cntn6-deficiency are clearly warranted.

Molenhuis et al. (2016) analyzed the development of neurological phenotypes in Cntn4-deficient mice at various developmental ages through an extended SHIRPA battery of tests. This study also included assessments for ASD-related behaviors such as the juvenile social interaction test, three-chamber social interaction test, novel object investigation task and the Barnes maze; however, Cntn4-deficiency was not observed to affect ASD-related behaviors (Molenhuis et al., 2016). Compared to wild-type littermates, Cntn4-deficient mice did not show any further changes in grooming behavior, social interaction, sensorimotor coordination, or cognitive flexibility (Molenhuis et al., 2016). No defects in olfaction were observed in Cntn4-deficient mice, which is unexpected since Cntn4 has been found to play a role in the establishment of odor maps (Kaneko-Goto et al., 2008). However, Cntn4-deficient mice do demonstrate enhanced spatial learning in the Barnes maze and a consistently increased startle response to auditory stimuli at high amplitudes (Molenhuis et al., 2016). Based on these findings, Molenhuis et al. (2016) concluded that Cntn4-deficiency does not affect ASD-specific phenotypes, but rather results in subtle non-ASD-specific changes in responsiveness to stimuli and cognitive performance. Notably, hyper-responsiveness to acoustic stimuli is a phenotype that has been associated with ASD and other neuropsychiatric disorders (Baranek et al., 2013; Green et al., 2013; Acevedo et al., 2018; Dakopolos and Jahromi, 2019; Hornix et al., 2019).

Overall, the lack of any strong phenotypes in Cntn4-deficient mice supports the idea that Cntn4 deficiency may be compensated for by other neuronal IgCAMs. As discussed in sections “Axon Guidance, Neurite Outgrowth, and Neuronal Migration” and “Synaptic Transmission and Plasticity,” Cntn4 regulates neurite outgrowth, axon guidance, dendritic spine morphology, and cell-surface neurotransmitter receptor expression. This discrepancy between the subtle behavior reported and phenotypes observed in *ex vivo* or *in vitro* cultures suggest other neuronal IgCAMs exhibit a degree of functional redundancy that can compensate for deficits in neuronal migration and neurite outgrowth. Indeed, Cntn4 deficiency is mainly reported to only cause mild abnormalities in gross cortical development and axon guidance in the olfactory bulb. Similarly, the effects of Chl1 and Cntn6 deficiency on neurite outgrowth are more pronounced within primary neuronal cultures, and milder phenotypes are observed in animal models. Studies in other CAMs, such as Neural Cell Adhesion Molecule 1 (Ncam1), L1 Cell Adhesion Molecule (L1cam) and several members of the Cadherin family have also indicated similar findings (reviewed in Hortsch, 1996; Colman and Filbin, 1999; Halbleib and Nelson, 2006; Dalva et al., 2007; Niessen et al., 2011; Lin et al., 2016). Ncam1- and L1cam-deficiency results in pronounced defects in neuronal migration and neurite outgrowth in culture, however, only subtle neuronal migration and behavior phenotypes are observed in Ncam1- and L1cam-knockout mice (Hortsch, 1996; Colman and Filbin, 1999). This may explain the highly variable severity of phenotypes in humans caused by CNVs in the 3p26.3 region and further suggests that multiple neuronal IgCAMs and their signaling pathways may interact with one another. When one gene is disrupted, others may act to suppress the behavioral phenotype by compensating for the loss of function.

Considering the region-specific expression patterns of *Chl1*, *Cntn6*, and *Cntn4*, such as in the hippocampus (discussed in section “Spatiotemporal Expression Patterns of CHL1, CNTN6, and CNTN4”) (Figure 5A), how can shared signaling pathways exist between these neuronal IgCAMs? For example, if certain axon guidance deficits in the hippocampus could be compensated for by different combinations of these neuronal IgCAMs, this can only occur in regions where co-expression of these neuronal IgCAMs does exist, e.g., in the CA1 and CA3 (Figure 5A). Yet, other regions remain unaccounted for, e.g., the CA2 and DG only express *Cntn4* and *Chl1*, respectively (Figure 5A), so how can common signaling pathways exist in these regions in order to compensate for axon guidance deficits? It is important to consider two factors: (1) the high structural similarity and shared binding partners among the Contactin family members (Shimoda and Watanabe, 2009; Bouyain and Watkins, 2010a); and (2) the capability of Chl1 to form large complexes through heterodimerization in *cis* and *trans* with other neuronal IgCAMs of the Contactin and CASPR families (Ashrafi et al., 2014). Other members of the L1, Contactin, and CASPR families should also be considered to be participating in regulating compensatory effects as a high degree of functional redundancy may exist between these neuronal IgCAMs. Some of these family members demonstrate region-specific expression as well. For example, *Cntn3* is expressed in the CA2 and not the DG, whilst *Cntn5*, *Caspr1*, *Caspr5*, and *L1cam* are expressed in the DG and not the CA2 region (©2015 Allen Institute for Brain Science; Hochgerner et al., 2018). Certain genes, such as *Cntn1*, *Caspr2*, *Caspr5*, and *Nrcam* are also commonly expressed in both the CA2 and DG (©2015 Allen Institute for Brain Science; Hochgerner et al., 2018). As interactors of Chl1 (Ashrafi et al., 2014), Cntn5 and Nrcam may initiate signaling pathways to compensate for Chl1 deficiency in the DG. Additionally, Cntn3 and Cntn5 are particularly interesting as, similar to Cntn6 and Cntn4, they are reported to interact with PTPRG to regulate neurite outgrowth (Bouyain and Watkins, 2010a,b). Notably, PTPRG is expressed in the CA1, CA2, CA3, and DG (©2015 Allen Institute for Brain Science; Hochgerner et al., 2018) – it is possible that interactors shared among these different neuronal IgCAMs such as PTPRG act as hub proteins to facilitate axon guidance in regions such as the hippocampus, allowing for compensation during instances where the expression or function of certain neuronal IgCAMs are disrupted. Therefore, not every member of the L1 and Contactin families need to be expressed in all cells of the hippocampus – rather, alternate family members form complex networks of interactomes to compensate for axon guidance deficits. In fact, the specific blend of these molecules in different neurons may bestow a combinatorial code upon different populations of neurons. It would also be pertinent to monitor the expression of other neuronal IgCAMs in Chl1-, Cntn6-, and Cntn4-deficient models to see if other family members are being upregulated to compensate for each other’s deficiency. Examples of this have been observed in other ASD models such as the Shank3 mutant mouse, where upregulation of Shank1 and Shank2 are observed to partially compensate for synaptic defects (Zhou et al., 2016). However, shared interactions such as PTPRG between the Contactins also raise the question of whether certain neuronal IgCAMs act as core components for axon guidance regulation and if others exhibit higher degrees of functional redundancy? At this point further research is still required to answer this question.

## Future Directions

Our understanding of the function of neuronal IgCAMs, *CHL1, CNTN6*, and *CNTN4*, encoded within the 3p26.3 region in neurodevelopment has greatly advanced over recent decades. Evidence has shown a genetic link between these genes, Del3p and ASD. In particular, there are specific CNVs reported in these genes (Supplementary Table 1). Through clinical reports and patient genetics, we are beginning to understand the link between CNVs in *CHL1, CNTN6*, and *CNTN4*, and the severity of Del3p and ASD phenotypes. CNVs occurring in these neuronal IgCAMs may affect the severity of ASD phenotypes through multiple molecular pathways. We hypothesize that disruption or imbalance of these pathways can contribute toward the ASD phenotype. However, the high variability of these CNVs makes it difficult to currently predict the effects of these mutations on gene dosage and their consequence on neurodevelopment. Further studies should aim to establish a link between CNV length, magnitude and location within a gene with the severity of ASD phenotypes reported in the patient in order to further elucidate genotype-phenotype relationships.

Disrupting the functions of CHL1, CNTN6, and CNTN4 and their signaling pathways may contribute to ASD via three main routes. Firstly, these neuronal IgCAMs participate in signaling pathways that regulate neurogenesis and neuronal survival. Chl1 induces activation of the PI3K, MAPK, and hedgehog signaling pathways (Nishimune et al., 2005; Katic et al., 2017), and Cntn6 modulates neuronal apoptosis by inhibiting the neurotoxic effects of Lphn1 (Zuko et al., 2016a). Cntn6 modulates activity of Lphn1 by direct binding, however, in regions of the brain Lphn1 may be regulated by other binding partners, since Cntn6 has a range of interactors (a common theme in the Contactin family) (Zuko et al., 2013). Secondly, these neuronal IgCAMs have been shown to interact with each other and with similar binding partners in the context of axon guidance, neuritogenesis and synapse formation likely participating in common signaling pathways and sequestering each other’s binding partners (Figure 5B). Chl1-Cntn6 interactions are important in regulating dendritic morphology (Ye et al., 2008), and Cntn6 and Cntn4 interact with Ptprg to promote neurite extension (Bouyain and Watkins, 2010b; Cottrell et al., 2011; Mercati et al., 2013). Additionally, CHL1 and CNTN4 may regulate axon guidance and arborization via direct heterodimerization and perhaps an overlapping pathway of APP processing (Osterfield et al., 2008; Osterhout et al., 2015; Ou-Yang et al., 2018). CNTN4 is shown to be an extracellular binding partner of the E1 domain of APP, although this interaction leaves the Ig domains of CNTN4 available for other binding partners. This is important since not only is APP linked with neurodegeneration but also neurodevelopment (Steinbach et al., 1998; Magara et al., 1999; Osterfield et al., 2008). Furthermore, the E1 domain of APP is important for neural stem cell differentiation (Ohsawa et al., 1999), synaptogenesis (Morimoto, 1998), and neurite outgrowth (Small et al., 1994; Ohsawa et al., 1997). The CNTN4-APP interaction has been observed in the olfactory bulb and retinal axons of the retinotectal system, which are regions where the development of axon–axon and axon-target contacts is crucial (Osterhout et al., 2015). Alterations in CNTN4 expression have been implicated to negatively affect the proteolytic processing of APP, which contributes to impairments in axon guidance and synaptic plasticity, reduced neuronal survival, and ultimately results in cognitive impairments (Bamford et al., 2020). Finally, deficiencies in CHL1, CNTN6, and CNTN4 may impair the formation and stabilization of synapses due to their role as synaptic adhesion molecules. Dysregulation of CHL1, CNTN6, and CNTN4 signaling is observed to alter synaptic function, causing impairments in synaptic plasticity and shifting the E/I balance in neuronal circuits. CHL1 and CNTN6 in particular play important roles in vesicular recycling at the presynapse (Leshchyns’ka et al., 2006; Sakurai et al., 2009, 2010; Andreyeva et al., 2010), and their deficiency alters both glutamatergic and GABAergic transmission and causes synaptic loss (Nikonenko et al., 2006; Sakurai et al., 2009, 2010; Schmalbach et al., 2015; Zuko et al., 2016b). CNTN4 deficiency is also observed to alter cell-surface expression of neurotransmitter receptors (Heise et al., 2018). Taken altogether, we hypothesize that disruptions to CHL1, CNTN6, and CNTN4 pathways impacts neurogenesis, neuronal survival and axon guidance. Disruptions to these neuronal IgCAMs can also lead to impairments in synaptic function by directly disrupting synapse formation and stabilization, or through altering synaptic transmission and plasticity. These impairments in turn can contribute to the cognitive and behavior phenotypes observed in Del3p and ASD.

Evidence from animal studies have also shown a potential involvement of these neuronal IgCAMs in the development of ASD-associated behaviors (Table 2), but importantly suggest that the deficiency of these molecules can be compensated for by other neuronal IgCAMs. The lack of strong behavioral phenotypes, as opposed to the pronounced defects in neuronal migration and neuritogenesis in culture, indicate that loss of function in CHL1, CNTN6, and CNTN4 may be compensated for by other neuronal IgCAMs which act to suppress the behavior phenotype. To better understand how these genes interact with one another, multi-gene models may be more appropriate than single-gene knockout studies. We hypothesize that a more severe phenotype will be observed when all three neuronal IgCAMs encoded in the 3p26.3 region are disrupted. A model in which all three genes have been disrupted will help to increase our understanding of how defects in multiple genes cause developmental syndromes such as Del3p. Such a model will also allow for further investigation into the formation of neuronal IgCAM complexes in different brain regions and the role they play in neurodevelopment.

## Author Contributions

AO-A: concept, research design, editing figures and artwork, and manuscript writing and editing. JG: research, generating figures and artwork, and manuscript writing. RB: formatting corrections and manuscript writing and editing. JB: critical input and manuscript editing. All authors read and approved the final manuscript.

## Conflict of Interest

The authors declare that the research was conducted in the absence of any commercial or financial relationships that could be construed as a potential conflict of interest.
